# The emerging role of GLP-1 receptor agonists in treating or preventing cancer

**DOI:** 10.20517/cdr.2024.116

**Published:** 2024-12-07

**Authors:** David J. Benjamin, Daniel D. Von Hoff

**Affiliations:** ^1^Department of Medical Oncology, Hoag Family Cancer Institute, Newport Beach, CA 92663, USA.; ^2^Molecular Medicine Division, Translational Genomics Research Institute, Phoenix, AZ 85004, USA.

**Keywords:** GLP-1 receptor agonist, cancer, prevention, resistance

## Abstract

With the growing incidence of obesity-related malignancies, glucagon-like peptide-1 (GLP-1) receptor agonists represent an intriguing potential clinical avenue for cancer prevention and treatment. Population-based data suggest that individuals who have taken GLP-1 receptor agonists have a decreased incidence of obesity-related cancers. Moreover, *in vivo* and *in vitro* studies have demonstrated the antitumor activity of these agents independent of other antineoplastic therapeutics. Additionally, other pre-clinical studies have shown that GLP-1 receptor agonists may help overcome resistance to chemotherapy-refractory cancer cells, thus demonstrating a plausible role in cancer treatment. Randomized controlled trials utilizing GLP-1 receptor agonists in both cancer prevention and treatment may allow for a better understanding of the role of these agents in modern oncology.

Glucagon-like peptide-1 (GLP-1) receptor agonists treat type 2 diabetes (T2D) and obesity through mechanisms including inhibition of glucagon secretion, food intake, and gastric emptying, as well as stimulation of insulin secretion^[1]^. As obesity is a risk factor for at least thirteen malignancies such as pancreatic and colon cancer through dysregulation of insulin and hormonal signaling pathways, the incidence of obesity-associated cancers is anticipated to increase concurrently with rising rates of obesity^[2]^. Consequently, there has been growing interest in evaluating GLP-1 receptor (GLP-1R) agonists for the prevention or treatment of cancers, particularly those associated with obesity.

GLP-1R expression has been found in several tissues ranging from the skin, nervous system, pancreas, gastrointestinal tract, and prostate^[3]^. In fact, GLP-1R expression has been found to be co-localized with P504S, a prostate cancer marker, in human prostatectomy specimens. Both *in vitro* and *in vivo* experiments utilizing GLP-1R agonist exendin-4 demonstrated decreased proliferation of prostate cancer cell lines by inhibiting the extracellular signaling-regulated kinase/mitogen-activated protein kinase (ERK/MAPK) pathway, independent of the androgen receptor axis^[4]^. In addition, GLP-1R agonists have been demonstrated to affect the obesity-dependent and -independent development of hepatocellular carcinoma by acting on multiple pathways including JNK, PI3K/Akt/mTOR, and EGFR-STAT3; however, it is unknown whether GLP-1R agonist activity in hepatocellular carcinoma is due to minimizing metabolic risk factors such as obesity and insulin resistance or from genuine antineoplastic properties - or both^[5]^. Similarly, *in vitro* studies of human ovarian cancer cell lines have demonstrated the ability of exendin-4 to activate caspases and modulate metalloproteinases, thereby promoting apoptosis and reducing cancer dissemination, respectively^[6]^. Other studies have demonstrated GLP-1R agonists may alter the gut microbiome, whose modulation has been linked to improved outcomes in early studies of renal cell carcinoma^[7,8]^. Finally, GLP-1R agonists exendin-4 and liraglutide have been shown to inhibit migration of cholangiocarcinoma cells by partly inhibiting epithelial-mesenchymal transition, and thus slowing down disease progression^[9]^. As such, through several potential mechanisms, pre-clinical data provide a rationale for the role of GLP-1R agonists in potentially treating several cancers.

Prospective clinical trials evaluating GLP-1R agonists in diabetes initially provided possible evidence for a role in cancer prevention. Exploratory secondary endpoint analysis of the LEADER trial demonstrated a reduced risk of developing prostate cancer in men treated with liraglutide compared with placebo [HR (hazard ratio) 0.54; 95%CI (confidence interval): 0.34-0.88]^[3]^. A subsequent retrospective study in the United States of 1,221,218 drug-naïve patients with T2D with or without obesity/overweight had a decreased risk of developing colorectal cancer (HR 0.75; 95%CI: 0.58-0.97) with GLP-1R agonists compared to non-insulin T2D medications and insulin (HR 0.56; 95%CI: 0.44-0.72) over a 15-year follow-up period^[10]^. An additional retrospective study of 1,651,452 individuals with T2D in the United States demonstrated that treatment with GLP-1R agonists in comparison to insulin was associated with a decreased incidence in 10 of 13 obesity-associated cancers including pancreatic (HR 0.41; 95%CI: 0.33-0.50), colorectal cancer (HR 0.54; 95%CI: 0.42-0.86), ovarian cancer (HR 0.52; 95%CI: 0.03-0.74), and multiple myeloma (HR 0.59; 95%CI: 0.44-0.77)^[11]^. These retrospective studies suggest a conceivable role for GLP-1R agonists in chemoprevention, particularly for high-risk individuals, such as those with obesity and/or T2D.

In addition to potential uses for cancer prevention, *in vivo* and *in vitro* studies have evaluated the use of GLP-1R agonists in combination with current standard-of-care antineoplastic therapeutics. GLP-1R expression was found to be lower in gemcitabine-resistant pancreatic cancer cell line PANC-GR compared to human pancreatic cancer cell line PANC-1^[12]^. Incubation of the gemcitabine-resistant cell line with GLP-1R agonist liraglutide led to increased GLP-1R expression. Moreover, liraglutide led to dose-dependent inhibition of pancreatic cancer cell growth possibly through apoptosis due to increased expression levels of caspase-3. Liraglutide also inhibited growth and increased apoptosis in the gemcitabine-resistant cell line, likely through NF-KB downregulation. In addition, liraglutide in combination with gemcitabine reduced tumor weight and volume in comparison to gemcitabine alone, thus suggestive of a synergistic effect warranting further study in the clinical setting.

Emerging data suggest a potential role of GLP-1R agonists in treating a multitude of conditions beyond obesity and T2D, ranging from hypertension to dementia^[13]^. Pre-clinical and retrospective clinical data strongly support further investigation of GLP-1R agonists in either cancer prevention or in combination with chemotherapy in treating cancer. For example, given no clear standard-of-care approach for treating biochemical recurrence of prostate cancer, GLP-1R agonists could be studied in combination with androgen deprivation therapy or as a single agent therapy given pre-clinical evidence of activity independent of the androgen receptor axis. Admittedly, similar promise for cardiovascular drugs such as HMG-CoA reductase inhibitors, aspirin, and metformin did not translate to clinical benefit in preventing or treating cancer when studied in prospective trials, possibly due to immortal time bias in observational studies^[14,15]^. As such, randomized controlled trials are required to determine the clinical benefit of GLP-1R agonists in cancer prevention and treatment.

## DECLARATIONS

### Copyright

© The Author(s) 2024.
